# Supplementary material for: Evolutionary Relationships and Range Evolution of Greenhood Orchids (Subtribe Pterostylidinae): Insights From Plastid Phylogenomics
Source: Front Plant Sci. 2022 Jun 29;13:912089. doi: 10.3389/fpls.2022.912089 (PMC9277221; doi:10.3389/fpls.2022.912089)
Supplement: Supplementary file 1 [file Data_Sheet_1.PDF]

frontiers

Supplementary Material S1

Plant material studied. [1]Jones and Clements (2002), with revisions from Jones (2015), Clements & Jones (2016), and Jones & Clements (2017). *GenBank accession numbers of previously published DNA sequences. Herbarium acronyms: CANB, Australian National Herbarium; CHR: Allan Herbarium; CNS: Australian Tropical Herbarium; NSW: National Herbarium of New South Wales.

Species	*Pterostylis* sections *sensu* Janes & Duretto (2010)	Species *sensu* Jones & Clements[1]	Voucher details	Sample identifier	Reference plastid data	Reference nuclear data	Ancestral range analysis
**Pterostylidinae**							
Achlydosa glandulosa (Schltr.) M.A.Clem. & D.L.Jones	n/a	*Achlydosa glandulosa* (Schltr.) M.A.Clem. & D.L.Jones	M.A.Clements 11237 (CANB 950931.1)	G03414	this study	this study	0
Pterostylis abrupta D.L.Jones	*Foliosae* G.Don	*Diplodium abruptum* (D.L.Jones) D.L.Jones & M.A.Clem	T.N.Hayashi 65 (CANB)	G04979	this study	this study	1
Pterostylis aciculiformis (Nicholls) M.A.Clem. & D.L.Jones	*Oligochaetochilus* (Szlach.) Janes & Duretto	*Oligochaetochilus aciculiformis* (Nicholls) Szlach.	G.Bradburn 40 (CANB 892536.1)	G05943	this study	this study	1
Pterostylis acuminata R.Br.	*Pterostylis* R.Br.	*Pterostylis acuminata* R.Br.	M.A.Clements 12014 (CANB 909845.1)	G03411	this study	-	1
Pterostylis aestiva D.L.Jones	*Foliosae* G.Don	*Diplodium aestivum* (D.L.Jones) D.L.Jones & M.A.Clem.	T.N.Hayashi 45 (CANB)	G05019	this study	this study	1
Pterostylis aff. *graminea* "Kauri"	*Pterostylis* R.Br.	*Pterostylis* aff. *graminea* "Kauri"	B.P.J.Molloy 124/99 (CHR 531784)	G03985	this study	-	1
Pterostylis agathicola D.L.Jones, Molloy & M.A.Clem.	*Pterostylis* R.Br.	*Pterostylis agathicola* D.L.Jones, Molloy & M.A.Clem.	P.J.deLange 3903 & R.O.Gardner 082/99 (CHR 518307)	G06188	this study	-	1
Pterostylis allantoidea R.S.Rogers	*Foliosae* G.Don	*Diplodium allantoideum* (R.S.Rogers) D.L.Jones & M.A.Clem.	M.A.Clements 10891a (CANB 644580.1)	G06192	this study	this study	1
Pterostylis alpina R.S.Rogers	*Pterostylis* R.Br.	*Pterostylis alpina* R.S.Rogers	ORG7428 (CANB 950938.1)	G05940	this study	this study	1
Pterostylis aneba D.L.Jones	*Pterostylis* R.Br.	*Pterostylis aneba* R.S.Rogers	P. Branwhite 392 (CANB 950935.1)	G03408	this study	this study	1
Pterostylis arenicola M.A.Clem. & J.Stewart	*Oligochaetochilus* (Szlach.) Janes & Duretto	*Oligochaetochilus arenicola* (M.A.Clem. & J.Stewart) Szlach.	M.Young 29 (CANB 950922.1)	G05951	this study	-	1
Pterostylis aspera D.L.Jones & M.A.Clem.	*Foliosae* G.Don	*Diplodium asperum* (D.L.Jones & M.A.Clem.) D.L.Jones & M.A.Clem.	C.J.French 6115 (CANB 666990.1)	G06194	this study	this study	1
Pterostylis atrans D.L.Jones	*Foliosae* G.Don	*Diplodium atrans* (D.L.Jones) D.L.Jones & M.A.Clem.	T.N.Hayashi 38 (CANB)	G04407	this study	this study	1
Pterostylis atrosanguinea (D.L.Jones & C.J.French) D.L.Jones & C.J.French	*Urochilus* (D.L.Jones & M.A.Clem.) Janes & Duretto	*Urochilus atrosanguineus* D.L.Jones & C.J.French	C.J.French 6120 (CANB 666994.1)	G06186	-	this study	0
Pterostylis banksii R.Br. ex A.Cunn.	*Pterostylis* R.Br.	*Pterostylis banksii* A.Cunn.	W.Parr 087/99 (CHR 518312)	G04072	this study	-	1
Pterostylis baptistii Fitzg.	*Pterostylis* R.Br.	*Pterostylis baptistii* Fitzg.	R.Crane 2088 (CANB 665689.1)	G04034	this study	-	1
Pterostylis barbata Lindl.	*Catochilus* Benth.	*Plumatichilos barbata* Szlach.	M.A.Clements 11966B (CANB 891201.1)	G01354	this study	this study	1

Species	*Pterostylis* sections *sensu* Janes & Duretto (2010)	Species *sensu* Jones & Clements[1]	Voucher details	Sample identifier	Reference plastid data	Reference nuclear data	Ancestral range analysis
Pterostylis bicornis D.L.Jones & M.A.Clem.	*Parviflorae* (Bent.) Janes & Duretto	*Petrorchis bicornis* (D.L.Jones & M.A.Clem.) D.L.Jones & M.A.Clem.	B.Dalyell ORG 4865 (CANB 673100.1)	G06427	this study	this study	1
Pterostylis boormanii Rupp	*Oligochaetochilus* (Szlach.) Janes & Duretto	*Oligochaetochilus boormanii* (Rupp) Szlach.	T.N.Hayashi 112 (CANB 950907.1)	G05972	this study	this study	1
Pterostylis brumalis L.B.Moore	*Foliosae* G.Don	*Diplodium brumale* (L.B.Moore) D.L.Jones, Molloy & M.A.Clem.	N.Pullman 078/99 (CHR 518302)	G06189	this study	this study	1
Pterostylis bryophila D.L.Jones	*Foliosae* G.Don	*Diplodium bryophilum* (D.L.Jones) D.L.Jones & M.A.Clem.	D.E.Murfet 1772 (CANB 676620.1)	G06413	this study	this study	1
Pterostylis bureaviana Schltr.	*Pterostylis* R.Br.	*Pterostylis bureaviana* Schltr.	M.A.Clements 11225 (CANB 664441.1)	G06209	this study	-	1
Pterostylis bureaviana Schltr.	*Pterostylis* R.Br.	*Pterostylis bureaviana* Schltr.	M.A.Clements 11180 (CANB 664439.1)	G06191	this study	this study	0
Pterostylis chlorogramma D.L.Jones & M.A.Clem.	*Squamatae* G.Don	*Bunochilus chlorogrammus* (D.L.Jones & M.A.Clem.) D.L.Jones & M.A.Clem.	T.N.Hayashi 97 (CANB 891065.1)	G05969	this study	this study	1
Pterostylis cobarensis M.A.Clem.	*Oligochaetochilus* (Szlach.) Janes & Duretto	*Oligochaetochilus cobarensis* (M.A.Clem.) Szlach.	G.Bradburn 27B (CANB 950910.1)	G05946	this study	this study	1
Pterostylis coccina Fitzg.	*Foliosae* G.Don	*Diplodium coccinum* (Fitzg.) D.L.Jones & M.A.Clem.	G.Bradburn 11 (CANB)	G04998	this study	this study	1
Pterostylis collina (Rupp) M.A.Clem. & D.L.Jones	*Foliosae* G.Don	*Diplodium collinum* (Rupp) M.A.Clem. & D.L.Jones	M.A.Clements 12419 (CANB)	G06196	this study	this study	1
Pterostylis concava D.L.Jones & M.A.Clem.	*Urochilus* (D.L.Jones & M.A.Clem.) Janes & Duretto	*Urochilus concavus* (D.L.Jones & M.A.Clem.) D.L.Jones & M.A.Clem.	C.J.French 6119 (CANB 666993.1)	G06212	this study	this study	1
Pterostylis concinna R.Br.	*Foliosae* G.Don	*Diplodium concinnum* (R.Br.) M.A.Clem. & D.L.Jones	M.A.Clements 12408 (CANB)	G06197	this study	this study	1
Pterostylis crassicaulis (D.L.Jones & M.A.Clem.) G.N.Backh.	*Hymenochilus* (D.L.Jones & M.A.Clem.) Janes & Duretto	*Hymenochilus crassicaulis* D.L.Jones & M.A.Clem.	M.A.Clements 12333 (CANB 950934.1)	G05968	this study	this study	1
Pterostylis cucullata R.Br.	*Pterostylis* R.Br.	*Pterostylis cucullata* R.Br.	R.Mawson ORG5141 (CANB 677099.1)	G06211	this study	this study	1
Pterostylis curta R.Br.	*Pterostylis* R.Br.	*Pterostylis curta* R.Br.	ORG7345 (CANB 950929.1)	G05071	-	this study	0
Pterostylis curta R.Br.	*Pterostylis* R.Br.	*Pterostylis curta* R.Br.	R.Crane 2042 (CANB 665695.1)	G03412	this study	this study	1
Pterostylis cycnocephala Fitzg.	*Hymenochilus* (D.L.Jones & M.A.Clem.) Janes & Duretto	*Hymenochilus cymbellus* Jones ined.	M.A.Clements 11916 (CANB 906118.1)	G02745	this study	this study	1
Pterostylis daintreana F.Muell. ex Benth.	*Pharochilum* (D.L.Jones & M.A.Clem.) Janes & Duretto	*Pharochilum daintreanum* (Benth.) D.L.Jones & M.A.Clem	T.N.Hayashi 76 (CANB 950936.1)	G05043	this study	this study	1

Species	*Pterostylis* sections *sensu* Janes & Duretto (2010)	Species *sensu* Jones & Clements[1]	Voucher details	Sample identifier	Reference plastid data	Reference nuclear data	Ancestral range analysis
Pterostylis decurva R.S.Rogers	*Foliosae* G.Don	*Diplodium decurvum* (R.S.Rogers) D.L.Jones & M.A.Clem.	M.A.Clements 10744 (CANB 664394.1)	G05977	this study	this study	1
Pterostylis dilatata A.S.George	*Foliosae* G.Don	*Diplodium dilatatum* (A.S.George) D.L.Jones & M.A.Clem.	C.J.French 6116 (CANB 666991.1)	G06193	this study	this study	1
Pterostylis dolichochila D.L.Jones & M.A.Clem.	*Foliosae* G.Don	*Diplodium dolichochilum* (D.L.Jones & M.A.Clem.) D.L.Jones & M.A.Clem.	M.A.Clements 11922 (CANB 909820.1)	G05959	this study	this study	1
Pterostylis dubia R.Br.	*Pterostylis* R.Br.	*Pterostylis dubia* R.Br.	M.A.Clements 11158 (CANB 681498.1)	G06185	this study	this study	1
Pterostylis ectypha (D.L.Jones & C.J.French) D.L.Jones & C.J.French	*Foliosae* G.Don	*Diplodium ectyphum* D.L.Jones & C.J.French	C.J.French 1570 (CANB 624821.1)	G02787	this study	this study	1
Pterostylis elegans D.L.Jones	*Foliosae* G.Don	*Diplodium elegans* (D.L.Jones) D.L.Jones & M.A.Clem.	T.N.Hayashi 72 (CANB)	G05029	this study	this study	1
Pterostylis erecta T.E.Hunt	*Pterostylis* R.Br.	*Pterostylis erecta* T.E.Hunt	M.A.Clements 11512 (CANB 882081.1)	G06198	this study	this study	1
Pterostylis erythroconcha M.A.Clem. & D.L.Jones	*Foliosae* G.Don	*Diplodium erythroconchum* (M.A.Clem. & D.L.Jones) D.L.Jones & M.A.Clem.	M.Young 17 (CANB 950923.1)	G05942	this study	this study	1
Pterostylis excelsa M.A.Clem.	*Oligochaetochilus* (Szlach.) Janes & Duretto	*Oligochaetochilus excelsus* (M.A.Clem.) Szlach.	M.A.Clements 11889 (CANB 950919.1)	G02748	this study	this study	1
Pterostylis falcata R.S.Rogers	*Pterostylis* R.Br.	*Pterostylis falcata* R.S.Rogers	T.N.Hayashi 25 (CANB 950906.1)	G04423	this study	this study	1
Pterostylis foliacea (D.L.Jones) D.L.Jones	*Catochilus* Benth.	*Plumatichilos foliaceus* D.L.Jones	M.A.Clements 11896 (CANB 906103.1)	G02746	this study	this study	1
Pterostylis foliata Hook.f.	*Pterostylis* R.Br.	*Pterostylis gracilis* Nicholls	M.A.Clements 11797 (CANB 906077.1)	G06207	this study	this study	1
Pterostylis furva (D.L.Jones) D.L.Jones	*Parviflorae* (Bent.) Janes & Duretto	*Speculantha furva* D.L.Jones	T.N.Hayashi 54 (CANB 950913.1)	G05034	this study	this study	1
Pterostylis gibbosa R.Br.	*Oligochaetochilus* (Szlach.) Janes & Duretto	*Oligochaetochilus gibbosus* (R.Br.) Szlach.	M.A.Clements 11039 (CANB 655142.1)	G01273	this study	this study	1
Pterostylis grandiflora R.Br.	*Foliosae* G.Don	*Diplodium grandiflorum* (R.Br.) D.L.Jones & M.A.Clem	M.A.Clements 12010 (CANB 909841.1)	G04878	this study	-	1
Pterostylis hamata Blackmore & Clemesha	*Oligochaetochilus* (Szlach.) Janes & Duretto	*Oligochaetochils hamatus* (Blackmore & Clemesha) Szlach.	G.Bradburn 25 (CANB 950909.1)	G05980	this study	this study	1
Pterostylis hildae Nicholls	*Pterostylis* R.Br.	*Pterostylis hildae* Nicholls	B.Dalyell ORG3471 (CANB 629210.1)	G04114	this study	this study	1
Pterostylis laxa Blackmore x *revoluta* R.Br.	*Foliosae* G.Don	*Diplodium laxum* (Blackmore) D.L.Jones & M.A.Clem. x *revolutum* (R.Br.) D.L.Jones & M.A.Clem.	M.A.Clements 12093 (CANB)	G05996	this study	this study	1
Pterostylis leptochila M.A.Clem. & D.L.Jones	*Oligochaetochilus* (Szlach.) Janes & Duretto	*Oligochateochilus leptochilus* (M.A.Clem. & D.L.Jones) Szlach.	M.A.Clements 12235 & L. Nauheimer (CANB)	G05964	this study	this study	1
Pterostylis lineata (D.L.Jones) G.N.Backh.	*Squamatae* G.Don	*Bunochilus lineatus* D.L.Jones	D.L.Jones 16514 (CANB 607105.1)	G05978	this study	this study	1

Species	*Pterostylis* sections *sensu* Janes & Duretto (2010)	Species *sensu* Jones & Clements[1]	Voucher details	Sample identifier	Reference plastid data	Reference nuclear data	Ancestral range analysis
Pterostylis lingua M.A.Clem.	*Oligochaetochilus* (Szlach.) Janes & Duretto	*Oligochaetochilus linguus* (M.A.Clem.) Szlach.	N.Reiter ORG6356 (CANB)	G06202	this study	this study	1
Pterostylis longifolia R.Br.	*Squamatae* G.Don	*Bunochilus longifolius* (R.Br.) D.L.Jones & M.A.Clem.	M.A.Clements 11755 (CANB 751196.1)	G04968	this study	this study	1
Pterostylis major (D.L.Jones) G.N.Backh.	*Squamatae* G.Don	*Bunochilus major* D.L.Jones	T.N.Hayashi 83 (CANB 891072.1)	G05947	this study	this study	1
Pterostylis melagramma D.L.Jones	*Squamatae* G.Don	*Bunochilus melagrammus* (D.L.Jones) D.L.Jones & M.A.Clem.	M.A.Clements 10738 (CANB 664392.1)	G04077	this study	this study	1
Pterostylis metcalfei D.L.Jones	*Foliosae* G.Don	*Diplodium metcalfei* (D.L.Jones) D.L.Jones & M.A.Clem.	T.N.Hayashi 82 (CANB)	G05979	this study	this study	1
Pterostylis mitchellii Lindl.	*Oligochaetochilus* (Szlach.) Janes & Duretto	*Oligochaetochilus mitchellii* (Lindl.) Szlach.	G.Bradburn 24 (CANB 950916.1)	G05953	this study	this study	1
Pterostylis monticola D.L.Jones	*Pterostylis* R.Br.	*Pterostylis monticola* D.L.Jones	T.N.Hayashi 18 (CANB 950904.1)	G04391	this study	this study	1
Pterostylis multiflora (D.L.Jones) G.N.Backh.	*Parviflorae* (Bent.) Janes & Duretto	*Speculantha multiflora* D.L.Jones	T.N.Hayashi 47 (CANB 950905.1)	G05016	this study	this study	1
Pterostylis nana R.Br.	*Foliosae* G.Don	*Diplodium clavigerum* (Fitzg.) D.L.Jones & M.A.Clem.	G.Bradburn 49 (CANB 950915.1)	G06206	this study	this study	1
Pterostylis nutans R.Br.	*Pterostylis* R.Br.	*Pterostylis nutans* R.Br.	M.A.Clements 12119 (CANB 906133.1)	G04366	this study	this study	1
Pterostylis nutans R.Br.	*Pterostylis* R.Br.	*Pterostylis hispidula* Fitzg.	K.Schulte 116 (CANB 950908.1)	G00238	this study	this study	0
Pterostylis obtusa R.Br.	*Foliosae* G.Don	*Diplodium obtusum* (R.Br.) D.L.Jones & M.A.Clem.	G.Bradburn 14 (CANB)	G05024	this study	this study	1
Pterostylis oreophila Clemesha	*Pterostylis* R.Br.	*Pterostylis oreophila* Clemesha	M.A.Clements 12335 (CANB)	G05966	this study	this study	1
Pterostylis pedoglossa Fitzg.	*Foliosae* G.Don	*Diplodium pedoglossum* (Fitzg.) M.A.Clem. & D.L.Jones	T.N.Hayashi 78 (CANB)	G05044	this study	this study	1
Pterostylis pedunculata R.Br.	*Pterostylis* R.Br.	*Pterostylis pedunculata* R.Br.	M.A.Clements 12116 (CANB 950914.1)	G04387	this study	-	1
Pterostylis porrecta D.L.Jones, Molloy & M.A.Clem.	*Pterostylis* R.Br.	*Pterostylis porrecta* D.L.Jones, Molloy & M.A.Clem.	P.J.deLange & J.W.D.Sawyer 149/99 (CHR 531809)	G06190	this study	-	1
Pterostylis prasina (D.L.Jones) G.N.Backh.	*Squamatae* G.Don	*Bunochilus prasinus* D.L.Jones	T.N.Hayashi 104 (CANB 950927.1)	G05933	this study	this study	1
Pterostylis psammophila (D.L.Jones) R.J.Bates	*Oligochaetochilus* (Szlach.) Janes & Duretto	*Oligochaetochilus psammophilus* D.L.Jones	M.Young 22 (CANB 950921.1)	G05967	this study	this study	1
Pterostylis pulchella Messmer	*Foliosae* G.Don	*Diplodium pulchellum* (Messmer) D.L.Jones & M.A.Clem.	H.M.E. Richards & R. Datodi 617 (CANB 950933.1)	G04872	this study	this study	1
Pterostylis pusilla R.S.Rogers	*Oligochaetochilus* (Szlach.) Janes & Duretto	*Oligochaetochilus pusillus* (R.S.Rogers) Szlach.	M.A.Clements 11872 (CANB 950920.1)	G02747	this study	-	1
Pterostylis pyramidalis Lindl.	*Foliosae* G.Don	*Diplodium pyramidalis* Lindl.) D.L.Jones & M.A.Clem.	M.A.Clements 11940B (CANB 891175.1)	G01115	-	this study	0

Species	*Pterostylis* sections *sensu* Janes & Duretto (2010)	Species *sensu* Jones & Clements[1]	Voucher details	Sample identifier	Reference plastid data	Reference nuclear data	Ancestral range analysis
Pterostylis recurva Benth.	*Stamnorchis* (D.L.Jones & M.A.Clem.) Janes & Duretto	*Stamnorchis recurva* (Benth.) D.L.Jones & M.A.Clem.	M.A.Clements 11938 (CANB 891173.1)	G01355	this study	this study	1
Pterostylis repanda (M.A.Clem. & D.L.Jones) J.M.H.Shaw	*Foliosae* G.Don	*Diplodium repandum* M.A.Clem. & D.L.Jones	D.L.Jones 15577 (CBG 9908875.1)	G04883	this study	this study	1
Pterostylis repanda (M.A.Clem. & D.L.Jones) J.M.H.Shaw	*Foliosae* G.Don	*Diplodium repandum* M.A.Clem. & D.L.Jones	M.A.Clements 11213 (CANB 664440.1)	G06210	this study	this study	0
Pterostylis revoluta R.Br.	*Foliosae* G.Don	*Diplodium revolutum* (R.Br.) D.L.Jones & M.A.Clem.	G.Bradburn 12 (CANB 950911.1)	G04997	this study	this study	1
Pterostylis robusta R.S.Rogers	*Foliosae* G.Don	*Diplodium robustum* (R.S.Rogers) D.L.Jones & M.A.Clem.	M.A.Clements 11890 (CANB 906098.1)	G03422	this study	this study	1
Pterostylis roensis M.A.Clem. & D.L.Jones	*Oligochaetochilus* (Szlach.) Janes & Duretto	*Oligochaetochilus roensis* (M.A.Clem. & D.L.Jones) Szlach.	M.A.Clements 10893b (CANB 644582.1)	G06204	this study	this study	1
Pterostylis rubescens (D.L.Jones) G.N.Backh.	*Parviflorae* (Bent.) Janes & Duretto	*Speculantha rubescens* D.L.Jones	M.A.Clements 11847 (CANB 906086.1)	G05012	this study	this study	1
Pterostylis rufa R.Br.	*Oligochaetochilus* (Szlach.) Janes & Duretto	*Oligochaetochilus rufus* (R.Br.) Szlach.	D.Herd ORG5019 (CANB 672882.1)	G06187	this study	this study	1
Pterostylis sanguinea D.L.Jones & M.A.Clem.	*Urochilus* (D.L.Jones & M.A.Clem.) Janes & Duretto	*Urochilus sanguineus* (D.L.Jones & M.A.Clem.) D.L.Jones & M.A.Clem.	T.N.Hayashi 100 (CANB 950926.1)	G05945	this study	this study	1
Pterostylis sargentii C.R.P.Andrews	*Urochilus* (D.L.Jones & M.A.Clem.) Janes & Duretto	*Ranorchis sargentii* (C.R.P.Andrews) D.L.Jones & M.A.Clem.	M.A.Clements 12308 & L.Nauheimer (CANB 950928.1)	G05973	this study	this study	1
Pterostylis setifera M.A.Clem., Matthias & D.L.Jones	*Oligochaetochilus* (Szlach.) Janes & Duretto	*Oligochaetochilus setifer* (M.A.Clem., Matthias & D.L.Jones) Szlach.	G.Bradburn 27 (CANB 950937.1)	G05938	this study	this study	1
Pterostylis setulosa (D.L.Jones & C.J.French) D.L.Jones & C.J.French	*Foliosae* G.Don	*Diplodium setulosum* D.L.Jones & C.J.French	M.A.Clements 10852 (CANB 644541.1)	G04435	this study	this study	1
Pterostylis smaragdyna D.L.Jones & M.A.Clem.	*Squamatae* G.Don	*Bunochilus smaragdynus* (D.L.Jones & M.A.Clem.) D.L.Jones & M.A.Clem.	T.N.Hayashi 98 (CANB 891075.1)	G05958	this study	this study	1
Pterostylis spec. *'pisinna'*	*Hymenochilus* (D.L.Jones & M.A.Clem.) Janes & Duretto	*Hymenochilus pisinnus* D.L.Jones ined.	M.Young 10a (CANB 950924.1)	G05970	this study	this study	1
Pterostylis spec. *'pisinna'*	*Hymenochilus* (D.L.Jones & M.A.Clem.) Janes & Duretto	*Hymenochilus pisinnus* D.L.Jones ined.	M.A.Clements 11920 (CANB 909819.1)	G02754	this study	this study	0
Pterostylis spec. *'protera'*	*Parviflorae* (Bent.) Janes & Duretto	*Speculantha* spec. *'protera'*	D.L.Jones ORG 7427 (CANB)	G05963	this study	-	1
Pterostylis squamata R.Br.	*Oligochaetochilus* (Szlach.) Janes & Duretto	*Oligochaetochilus squamatus* (R.Br.) Szlach.	T.N.Hayashi 30 (CANB)	G04429	this study	this study	1

Species	*Pterostylis* sections *sensu* Janes & Duretto (2010)	Species *sensu* Jones & Clements[1]	Voucher details	Sample identifier	Reference plastid data	Reference nuclear data	Ancestral range analysis
Pterostylis striata Fitzg.	*Foliosae* G.Don	*Diplodium striatum* (Fitzg.) D.L.Jones & M.A.Clem.	M.A. Clements 11782 (CANB)	G06214	this study	this study	1
Pterostylis tenuissima Nicholls	*Foliosae* G.Don	*Diplodium tenuissimum* (Nicholls) D.L.Jones & M.A.Clem.	M.Duncan ORG 5146 (CANB 677104.1)	G06199	this study	this study	1
Pterostylis terminalis (D.L.Jones & R.J.Bates) J.M.H.Shaw	*Oligochaetochilus* (Szlach.) Janes & Duretto	*Oligochaetochilus terminalis* D.L.Jones & R.J.Bates	M.A.Clements 11866 (CANB 950918.1)	G03417	this study	this study	1
Pterostylis torquata D.L.Jones	*Foliosae* G.Don	*Diplodium torquatum* (D.L.Jones) D.L.Jones & M.A.Clem.	T.N.Hayashi 81 (CANB)	G05981	this study	this study	1
Pterostylis truncata Fitzg.	*Foliosae* G.Don	*Diplodium truncatum* (Fitzg.) D.L.Jones & M.A.Clem.	G.Bradburn 13 (CANB)	G04996	this study	this study	1
Pterostylis tunstallii D.L.Jones & M.A.Clem.	*Squamatae* G.Don	*Bunochilus tunstallii* (D.L.Jones & M.A.Clem.) D.L.Jones & M.A.Clem.	M.A.Clements 11802 (CANB)	G06203	this study	this study	1
Pterostylis turfosa Endl.	*Catochilus* Benth.	*Plumatichilos turfosus* (Endl.) Szlach.	M.A.Clements 12309 & L. Nauheimer (CANB 950939.1)	G05949	this study	this study	1
Pterostylis valida (Nicholls) D.L.Jones	*Oligochaetochilus* (Szlach.) Janes & Duretto	*Oligochaetochilus validus* (Nicholls) D.L.Jones & M.A.Clem.	J.Whitfield ORG6159 (CANB 755628.1)	G06213	this study	this study	1
Pterostylis venosa Colenso	*Pterostylis* R.Br.	*Pterostylis venosa* Colenso	D.L.Jones s/n (CANB 787778.1)	G07751	-	this study	0
Pterostylis ventricosa (D.L.Jones) G.N.Backh.	*Parviflorae* (Bent.) Janes & Duretto	*Speculantha ventricosa* D.L.Jones	A.Stephenson ORG7309 (CANB)	G04340	this study	this study	1
Pterostylis vittata Lindl.	*Urochilus* (D.L.Jones & M.A.Clem.) Janes & Duretto	*Urochilus vittatus* (Lindl.) D.L.Jones & M.A.Clem.	M.A.Clements 11939 (CANB 891174.1)	G01353	this study	this study	1
Pterostylis williamsonii D.L.Jones	*Squamatae* G.Don	*Bunochilus williamsonii* (D.L.Jones) D.L.Jones & M.A.Clem.	M.A.Clements 10779A (CANB 891077.1)	G06218	this study	this study	1
Pterostylis xerophila M.A.Clem.	*Oligochaetochilus* (Szlach.) Janes & Duretto	*Oligochaetochilus xerophilus* (M.A.Clem.) Szlach.	M.A.Clements 11595 (CANB 882126.1)	G06201	this study	this study	1
**Outgroups**							
Adenochilus gracilis Hook.f.		*Adenochilus gracilis* Hook.f.	B.P.J.Molloy 174/00 (CHR 532785)	G04014	this study	this study	0
Anathallis obovata (Lindl.) Pridgeon & M.W.Chase		*Anathallis obovata* (Lindl.) Pridgeon & M.W.Chase	GenBank accession	NC043905*	NC043905*	-	0
Aphyllorchis anomala Dockrill		*Aphyllorchis anomala* Dockrill	K.Schulte 143 (CNS 144311.1)	G00168	this study	-	0
Apostasia wallichii R.Br.		*Apostasia stylidioides* (F.Muell.) Rchb.f.	K.Schulte 258 (CNS)	G06011	this study	-	0
Calypso bulbosa (L.) Oakes		*Calypso bulbosa* (L.) Oakes	GenBank accession	NC040980*	NC040980*	-	0
Cheirostylis notialis D.L.Jones		*Cheirostylis notialis* D.L.Jones	J.Moye ORG6978 (CANB 950940.1)	G03406	this study	-	0
Chloraea gavilu Lindl.		*Chloraea gavilu* Lindl.	Givnish et al. 2018	Givnish_2018	Givnish 2018	-	0
Chloraea gavilu Lindl.		*Chloraea gavilu* Lindl.	GenBank accession	FR832121*	-	FR832121*	0
Chloraea multiflora Lindl.		*Chloraea multiflora* Lindl.	GenBank accession	FR832119*	-	FR832119*	0

Species	Species *sensu* Jones & Clements[1]	Voucher details	Sample identifier	Reference plastid data	Reference nuclear data	Ancestral range analysis
Codonorchis lessonii (d'Urv.) Lindl.	*Codonorchis lessonii* (d'Urv.) Lindl.	E.Pisano & O.Dollenz 5832 (CANB 950903.1)	G05327	this study	this study	0
Coilochilus neocaledonicum Schltr.	*Coilochilus neocaledonicum* Schltr.	M.A.Clements 11248 (CANB 950932.1)	G05305	this study	-	0
Cooktownia robertsii D.L.Jones	*Cooktownia robertsii* D.L.Jones	L.J.Roberts (ORG 2113) (CANB 679460.1)	G06630	this study	-	0
Cypripedium japonicum Thunb.	*Cypripedium japonicum* Thunb.	GenBank accession	NC027227*	NC027227*	-	0
Danhatchia australis (Hatch) Garay & Christenson	*Danhatchia novaehollandiae* D.L.Jones & M.A.Clem.	P.H.Weston, W.A.Cherry & J. Stockard 3405 (NSW 870437)	G03415	this study	-	0
Dendrobium aduncum Lindl.	*Dendrobium aduncum* Lindl.	GenBank accession	NC038077*	NC038077*	-	0
Dendrobium flexicaule Z.H.Tsi, S.C.Sun & L.G.Xu	*Dendrobium flexicaule* Z.H.Tsi, S.C.Sun & L.G.Xu	GenBank accession	NC038076*	NC038076*	-	0
Disa bracteata Sw.	*Disa bracteata* Sw.	M.A.Clements & L.Nauheimer 12256 (CANB 906207.1)	G05322	this study	this study	0
Diuris sulphurea R.Br.	*Diuris sulphurea* R.Br.	M.A.Clements 12053 (CANB 908278.1)	G02433	this study	this study	0
Elleanthus sodiroi Schltr.	*Elleanthus sodiroi* Schltr.	GenBank accession	NC027266*	NC027266*	-	0
Epipactis palustris (L.) Crantz	*Epipactis palustris* (L.) Crantz	GenBank accession	NC041187*	NC041187*	-	0
Eucosia umbrosa D.L.Jones & M.A.Clem.	*Eucosia umbrosa* D.L.Jones & M.A.Clem.	D.L.Jones s.n. (CANB 676421.1)	G06632	this study	-	0
Eucosia viridiflora (Blume) M.C.Pace	*Goodyera viridiflora* (Blume) Blume	W.K.Harris 224 (CANB 950917.1)	G01321	this study	-	0
Eulophia graminea Lindl.	*Eulophia graminea* Lindl.	C.P.Brock 311 (CANB 596921.1)	G02766	this study	-	0
Gastrochilus calceolaris (Buch.-Ham. ex Sm.) D.Don	*Gastrochilus calceolaris* (Buch.-Ham. ex Sm.) D.Don	GenBank accession	NC042686*	NC042686*	-	0
Goodyera schlechtendaliana Rchb.f.	*Goodyera schlechtendaliana* Rchb.f.	M.A.Clements 12186 (CANB 950929.1)	G04898	this study	this study	0
Habenaria elongata R.Br.	*Pecteilis elongata* R.Br.	A.R.Field 3904 (CNS)	G03456	this study	this study	0
Hetaeria oblongifolia Blume	*Hetaeria oblongifolia* Blume	K.Schulte 150 (CNS 144275.1)	G00729	this study	-	0
Holcoglossum subulifolium (Rchb.f.) Christenson	*Holcoglossum subulifolium* (Rchb.f.) Christenson	GenBank accession	NC041519*	NC041519*	-	0
Neottia japonica (Blume) Szlach.	*Neottia japonica* (Blume) Szlach.	GenBank accession	NC041446*	NC041446*	-	0
Nervilia aragoana Gaudich.	*Nervilia aragoana* Gaudich.	L.Roberts ORG3787 (CANB 669943.1)	G03442	this study	this study	0
Paphiopedilum delenatii Guillaumin	*Paphiopedilum delenatii* Guillaumin	GenBank accession	NC041309*	NC041309*	-	0
Pendulorchis himalaica (Deb, Sengupta & Malick) Z.J.Liu, K.Wei Liu & X.J.Xiao	*Pendulorchis himalaica* (Deb, Sengupta & Malick) Z.J.Liu, K.Wei Liu & X.J.Xiao	GenBank accession	NC041513*	NC041513*	-	0
Peristylus banfieldii (F.M.Bailey) Lavarack	*Peristylus banfieldii* (F.M.Bailey) Lavarack	R.Collins 1158 (CANB 7904962)	G06628	this study	-	0
Phragmipedium longifolium (Rchb.f. & Warsz.) Rolfe	*Phragmipedium longifolium* (Rchb.f. & Warsz.) Rolfe	GenBank accession	NC028149*	NC028149*	-	0

Species	Species *sensu* Jones & Clements[1]	Voucher details	Sample identifier	Reference plastid data	Reference nuclear data	Ancestral range analysis
Rhomboda polygonoides (F.Muell.) Ormerod	*Rhomboda polygonoides* (F.Muell.) Ormerod	K.Schulte 249 (CNS 144274.1)	G00708	this study	-	0
Salacistis rubicunda (Blume) M.C.Pace	*Salacistis ochroleuca* (F.M.Bailey) M.A.Clem. & D.L.Jones	B.Gray 8333 (CANB 507310.1)	G06635	this study	-	0
Sobralia callosa L.O.Williams	*Sobralia callosa* L.O.Williams	GenBank accession	NC028147*	NC028147*	-	0
Spiranthes aestivalis (Poir.) Rich.	*Spiranthes aestivalis* (Poir.) Rich.	M.A.Clements 12199 (CANB 909010.1)	G05319	this study	this study	0
Spiranthes sinensis (Pers.) Ames	*Spiranthes australis* Lindl.	T.N.Hayashi 67	G04983	this study	this study	0
Townsonia viridis (Hook.f.) Schltr.	*Townsonia viridis* (Hook.f.) Schltr.	H.Wapstra ORG 4598 (CANB 670901.1)	G01314	this study	-	0
Vanilla aphylla Blume	*Vanilla aphylla* Blume	GenBank accession	NC035320*	NC035320*	-	0
Vanilla pompona Schiede	*Vanilla pompona* Schiede	GenBank accession	NC036809*	NC036809*	-	0
Zeuxine oblonga R.S.Rogers & C.T.White	*Zeuxine oblonga* R.S.Rogers & C.T.White	K.Schulte 181 (CNS 144329.1)	G00175	this study	this study	0
