# Supplementary material for: Evolutionary Relationships and Range Evolution of Greenhood Orchids (Subtribe Pterostylidinae): Insights From Plastid Phylogenomics
Source: Front Plant Sci. 2022 Jun 29;13:912089. doi: 10.3389/fpls.2022.912089 (PMC9277221; doi:10.3389/fpls.2022.912089)
Supplement: Supplementary file 3 [file Data_Sheet_3.PDF]

Supplementary Material S3

Divergence time estimations based on plastid and nuclear data.

Supplementary Material 3.1. Selection of the best fit models for divergence time estimation based on the 25 most parsimony informative plastid genes. Molecular clock and speciation models were evaluated using a posterior simulation-based analogue of Akaike's information criterion (AIC; Akaike, 1974), termed AICM (Raftery et al., 2007 as implemented in AICM Analyser included in BEASTv2.6.1. The best fit model for each dataset is highlighted in bold. The most parsimony informative plastid genes in descending order: *rpo*C2, *ycf*2, *rpo*B, *mat*K, *acc*D, *ndh*F, *rpo*C1, *ccs*A, *psa*B, *rps*16, *psa*A, *atp*A, *ndh*D, *rpo*A, *atp*B, *psb*B, *clp*P, *rps*2, *cem*A, *rps*3, *ndh*A, *psb*C, *atp*F, and *psb*A.

Dataset	Clock model	Model for speciation/extinction process	AICM
**Plastid**	pure birth (Yule)	uncorrelated relaxed lognormal clock	418029.1158
	pure birth (Yule)	strict clock	422354.0833
	birth death	uncorrelated relaxed lognormal clock	**418019.8344**
	birth death	strict clock	422336.9326
**Nuclear**	pure birth (Yule)	uncorrelated relaxed lognormal clock	**89144.3379**
	pure birth (Yule)	strict clock	89759.142
	birth death	uncorrelated relaxed lognormal clock	89161.9538
	birth death	strict clock	89689.3996

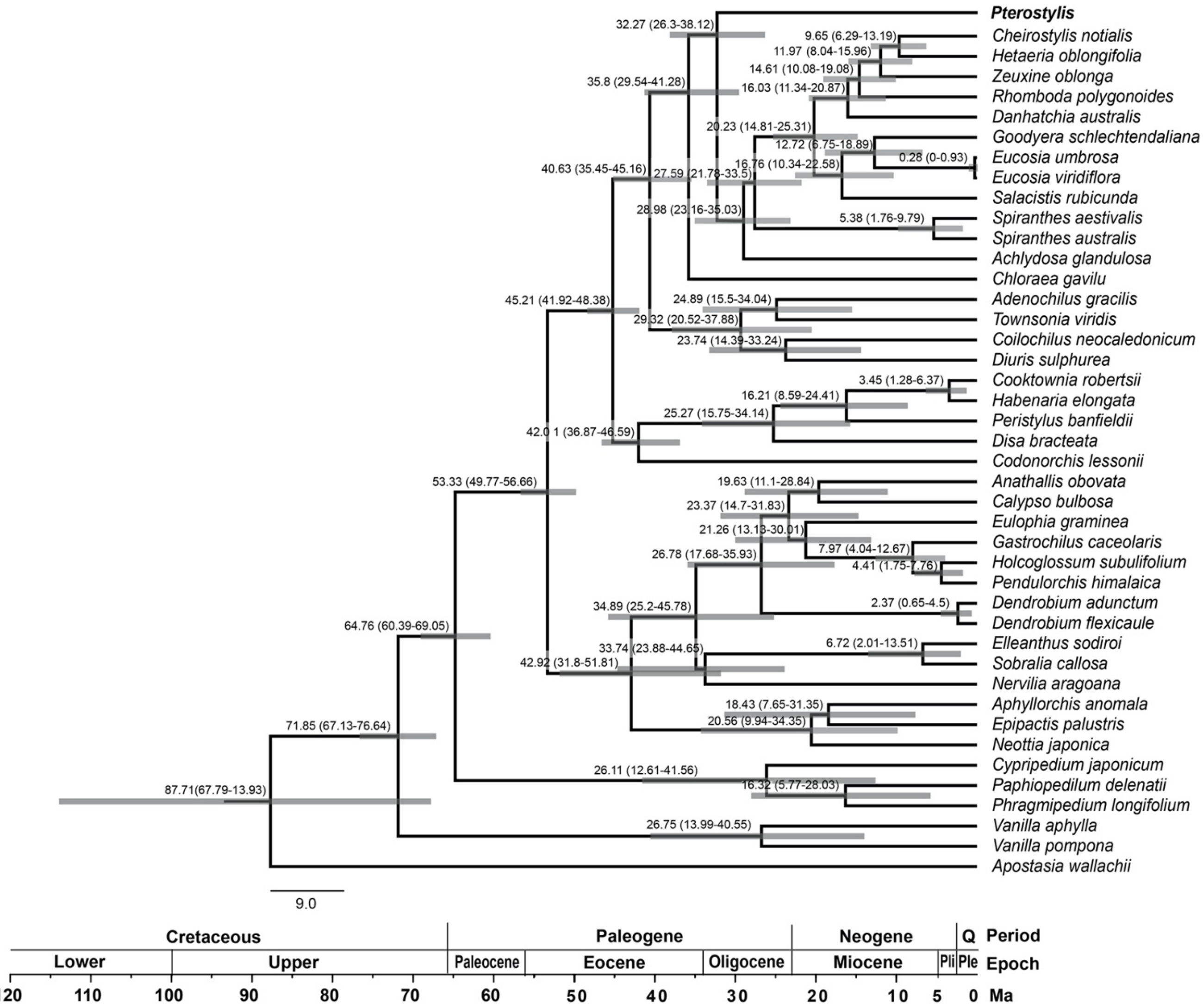

Supplementary Material 3.2. Chronogram showing divergence time estimations within Orchidaceae. Maximum clade credibility tree from Bayesian divergence time estimation based on 25 most informative plastid genes and an uncorrelated molecular clock model under the birth-death tree prior. Divergence times (Ma) are given at each node together with 95% highest posterior density (HDP) values indicated by grey bars and numbers in brackets.

Next page: Supplementary Material 3.3. Chronogram showing divergence times within Pterostylidinae *s.s.*. based on plastid data. Maximum clade credibility tree from Bayesian divergence time estimation based on 25 most informative plastid genes and an uncorrelated molecular clock model under the birth-death tree prior. Divergence times (Ma) are given at each node together with 95% highest posterior density (HDP) values indicated by grey bars and numbers in brackets.

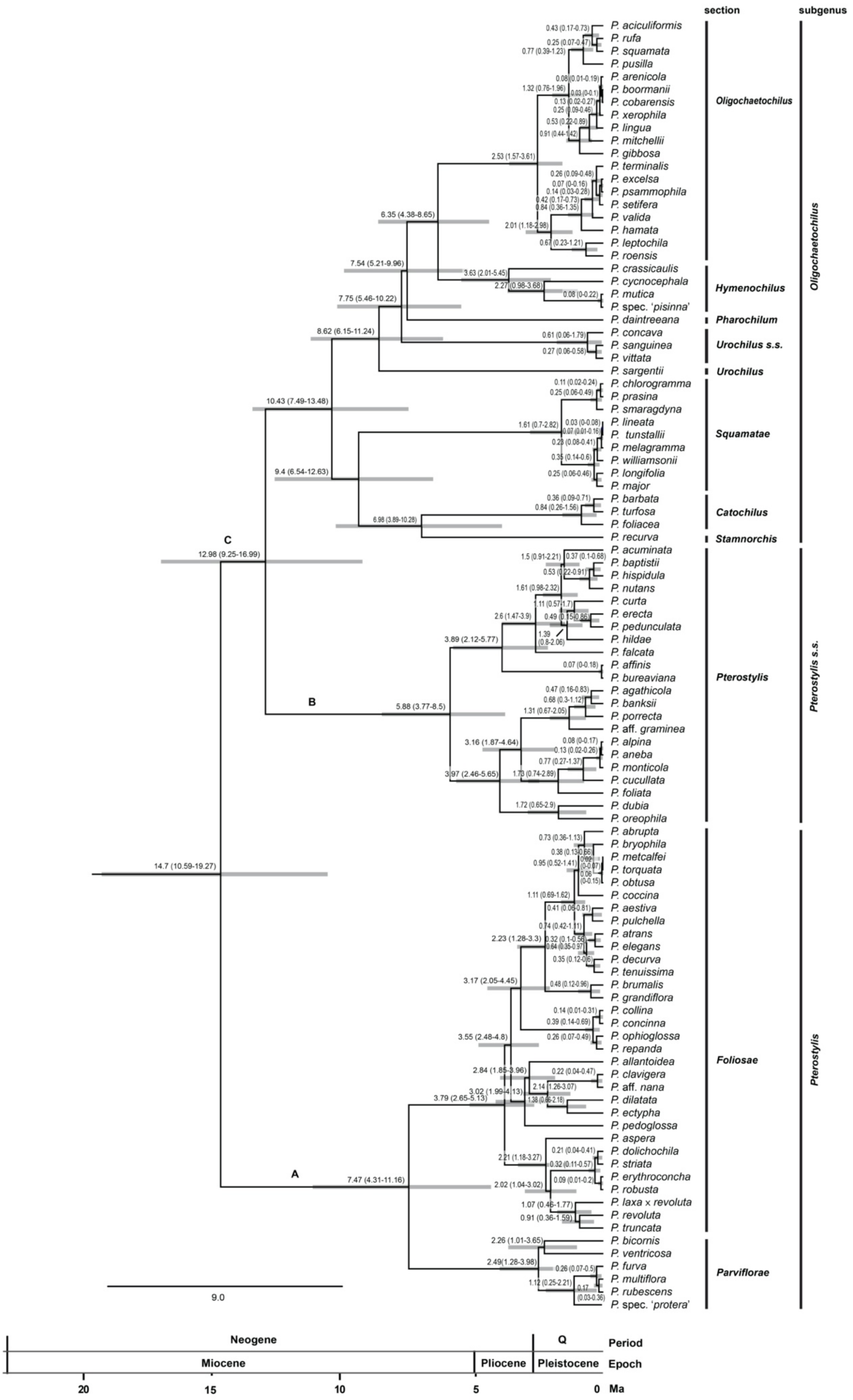
section
subgenus
P. aciculiformis
P. rufa
P. squamata
P. pusilla
P. arenicola
P. boormanii
P. cobarensis
P. xerophila
P. lingua
P. mitchellii
P. gibbosa
P. terminalis
P. excelsa
P. psammophila
P. setifera
P. valida
P. hamata
P. leptochila
P. roensis
P. crassicaulis
P. cycnocephala
P. mutica
P. spec. 'pisinna'
P. daintreeana
P. concava
P. sanguinea
P. vittata
P. sargentii
P. chlorogramma
P. prasina
P. smaragdyna
P. lineata
P. tunstallii
P. melagramma
P. williamsonii
P. longifolia
P. major
P. barbata
P. turfosa
P. foliacea
P. recurva
P. acuminata
P. baptistii
P. hispidula
P. nutans
P. curta
P. erecta
P. pedunculata
P. hildae
P. falcata
P. affinis
P. bureaviana
P. agathicola
P. banksii
P. porrecta
P. aff. graminea
P. alpina
P. aneba
P. monticola
P. cucullata
P. foliata
P. dubia
P. oreophila
P. abrupta
P. bryophila
P. metcalfei
P. torquata
P. obtusa
P. coccina
P. aestiva
P. pulchella
P. atrans
P. elegans
P. decurva
P. tenuissima
P. brumalis
P. grandiflora
P. collina
P. concinna
P. ophioglossa
P. repanda
P. allantoidea
P. clavigera
P. aff. nana
P. dilatata
P. ectypha
P. pedoglossa
P. aspera
P. dolichochila
P. striata
P. erythroconcha
P. robusta
P. laxa × revoluta
P. revoluta
P. truncata
P. bicornis
P. ventricosa
P. furva
P. multiflora
P. rubescens
P. spec. 'protera'
Oligochaetochilus
Hymenochilus
Pharochilum
Urochilus s.s.
Urochilus
Squamatae
Catochilus
Stamnorchis
Pterostylis
Foliosae
Parviflorae
Oligochaetochilus
Pterostylis s.s.
Pterostylis
C
12.98 (9.25-16.99)
10.43 (7.49-13.48)
9.4 (6.54-12.63)
8.62 (6.15-11.24)
7.75 (5.46-10.22)
7.54 (5.21-9.96)
6.35 (4.38-8.65)
B
5.88 (3.77-8.5)
14.7 (10.59-19.27)
A
7.47 (4.31-11.16)
9.0
Neogene
Q
Period
Miocene
Pliocene
Pleistocene
Epoch
20
15
10
5
0
Ma

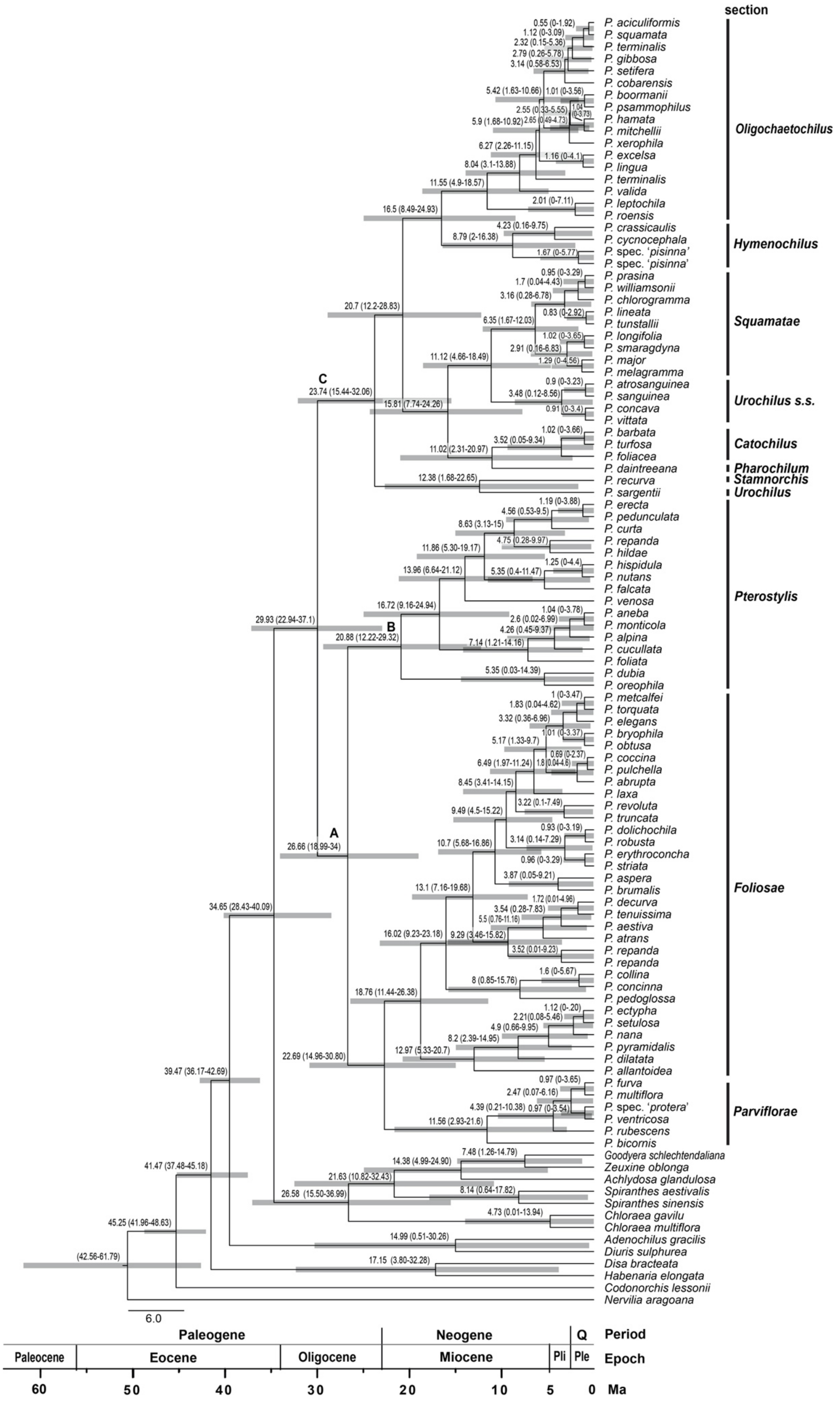
section
P. aciculiformis
P. squamata
P. terminalis
P. gibbosa
P. setifera
P. cobarensis
P. boormanii
P. psammophilus
P. hamata
P. mitchellii
P. xerophila
P. excelsa
P. lingua
P. terminalis
P. valida
P. leptochila
P. roensis
Oligochaetochilus
P. crassicaulis
P. cycnocephala
P. spec. 'pisinna'
P. spec. 'pisinna'
Hymenochilus
P. prasina
P. williamsonii
P. chlorogramma
P. lineata
P. tunstallii
P. longifolia
P. smaragdyna
P. major
P. melagramma
Squamatae
P. atrosanguinea
P. sanguinea
P. concava
P. vittata
Urochilus s.s.
P. barbata
P. turfosa
P. foliacea
Catochilus
P. daintreeana
Pharochilum
P. recurva
Stamnorchis
P. sargentii
Urochilus
P. erecta
P. pedunculata
P. curta
P. repanda
P. hildae
P. hispidula
P. nutans
P. falcata
P. venosa
P. aneba
P. monticola
P. alpina
P. cucullata
P. foliata
P. dubia
P. oreophila
Pterostylis
P. metcalfei
P. torquata
P. elegans
P. bryophila
P. obtusa
P. coccina
P. pulchella
P. abrupta
P. laxa
P. revoluta
P. truncata
P. dolichochila
P. robusta
P. erythroconcha
P. striata
P. aspera
P. brumalis
P. decurva
P. tenuissima
P. aestiva
P. atrans
P. repanda
P. repanda
P. collina
P. concinna
P. pedoglossa
P. ectypha
P. setulosa
P. nana
P. pyramidalis
P. dilatata
P. allantoidea
Foliosae
P. furva
P. multiflora
P. spec. 'protera'
P. ventricosa
P. rubescens
P. bicornis
Parviflorae
Goodyera schlechtendaliana
Zeuxine oblonga
Achlydosa glandulosa
Spiranthes aestivalis
Spiranthes sinensis
Chloraea gavilu
Chloraea multiflora
Adenochilus gracilis
Diuris sulphurea
Disa bracteata
Habenaria elongata
Codonorchis lessonii
Nervilia aragoana
A
B
C
6.0
Paleogene
Neogene
Q
Period
Paleocene
Eocene
Oligocene
Miocene
Pli
Ple
Epoch
60
50
40
30
20
10
5
0
Ma

Previous page: Supplementary Material S3.4. Chronogram showing divergence times within Pterostylidinae *s.s.* based on nuclear data. Maximum clade credibility tree from Bayesian divergence time estimation based on nuclear ribosomal RNA cistron and an uncorrelated molecular clock model under the Yule tree prior. Divergence times (Ma) are given at each node together with 95% highest posterior density (HDP) values indicated by grey bars and numbers in brackets.

Supplementary Material S3.5. Comparison of divergence time estimations of main lineages within Pterostylidinae *s.s.* derived from plastid and nuclear data. HDP: 95% highest posterior density.

	Divergence time estimate based on plastid data			Divergence time estimate based on nuclear data		
**Node**	**Ma**	**HDP range**	**epoch**	**Ma**	**HDP range**	**epoch**
Pterostylidinae s.s. stem	32.27	26.3-38.12	Oligocene	34.65	28.43-40.09	Oligocene
Pterostylidinae s.s. crown	14.7	10.59-19.27	mid Miocene	29.93	22.94-37.1	early Oligocene
Pterostylis Clade A crown	7.47	4.31-11.16	late Miocene	22.69	14.96-30.90	early Miocene
Pterostylis Clade B crown	5.88	3.77-8.5	late Miocene	20.88	12.22-29.32	early Miocene
Pterostylis Clade C crown	10.43	7.49-13.48	late Miocene	23.74	15.44-32.06	late Oligocene
P. sect. *Speculantha* stem	7.47	4.31-11.16	late Miocene	22.69	14.96-30.80	early Miocene
P. sect. *Speculantha* crown	2.49	1.28-3.98	early Pleistocene	11.56	2.93-21.6	late Miocene
P. sect. *Foliosae* stem	7.47	4.31-11.16	late Miocene	22.69	14.96-30.8	early Miocene
P. sect. *Foliosae* crown	3.79	2.65-5.13	early Pliocene	18.76	11.44-26.38	early Miocene
P. sect. *Pterostylis* stem	12.98	9.25-16.99	mid Miocene	29.93	22.94-37.1	early Oligocene
P. sect. *Pterostylis* crown	5.88	3.77-8.5	late Miocene	20.88	12.22-29.32	mid Miocene
P. sect. *Stamnorchis* stem	6.98	3.89-10.28	late Miocene	12.38	1.68-22.68	mid Miocene
P. sect. *Catochilus* stem	6.98	3.89-10.28	late Miocene	15.81	7.74-24.26	early Miocene
P. sect. *Catochilus* crown	0.84	0.26-1.56	early Pleistocene	10.02	2.31-20.97	late Miocene
P. sect. *Urochilus* s.s. stem	7.75	5.46-10.22	late Miocene	11.12	4.66-18.49	late Miocene
P. sect. *Urochilus* s.s. crown	0.61	0.06-1.79	mid Pleistocene	3.48	0.12-8.56	late Pleiocene
P. sect. *Squamatae* stem	9.4	6.54-12.63	late Miocene	11.12	4.66-18.49	late Miocene
P. sect. *Squamatae* crown	1.61	0.7-2.82	early Pleistocene	6.35	1.67-12.03	late Miocene
P. sect. *Hymenochilus* stem	6.35	4.38-8.65	late Miocene	16.5	8.49-24.93	early Miocene
P. sect. *Hymenochilus* crown	3.63	2.01-5.45	early Pliocene	8.79	2-16.38	late Miocene
P. sect. *Oligochaetochilus* stem	6.35	4.38-8.65	late Miocene	16.5	8.49-24.93	early Miocene
P. sect. *Oligochaetochilus* crown	2.53	1.57-3.61	late Pliocene	11.55	4.9-18.57	mid Miocene
P. sargentii stem	8.62	6.15-11.24	late Miocene	12.38	1.68-22.65	mid Miocene
