# Supplementary material for: Evolutionary Relationships and Range Evolution of Greenhood Orchids (Subtribe Pterostylidinae): Insights From Plastid Phylogenomics
Source: Front Plant Sci. 2022 Jun 29;13:912089. doi: 10.3389/fpls.2022.912089 (PMC9277221; doi:10.3389/fpls.2022.912089)
Supplement: Supplementary file 4 [file Data_Sheet_4.PDF]

Supplementary Material S4

Supplementary Material S4.1. Model comparison of three biogeographic models for ancestral range estimations in Pterostylidinae. Based on maximum clade credibility tree from divergence time estimations analysis under the birth-death tree prior and uncorrelated clock model based on 25 plastid loci. Log likelihoods (LnL), number of model parameters (n), Akaike Information Criterion values (AIC< Akaike 1973), and AIC weights (Akaike 1978) are given. The DEC model was determined as the best-fit model based on the lowest AIC score and thus was selected for ML ancestral range reconstruction in Pterostylidinae.

Model	LnL	n	AIC	$AIC_{weighted}$
DEC	-157.6	2	319.1	0.69
DIVALIKE	-172.4	2	348.9	0.28
BAYAREALIKE	-180.8	2	365.5	0.0082

References

Akaike, H. (1973). Information theory and an extension of the maximum likelihood principle. Proceedings of the Second International Symposium on Information Theory, e. B.N. Petrov, F. Caski. (Budapest, Akademiai Kiado), 267-281.

Akaike, H. (1978). On the likelihood of a time series model. The Statistician 27, 217.

Supplementary Material S4.2. Relative probabilities of range evolutionary scenarios in Pterostylidinae *s.s.* as inferred based on maximum likelihood under the dispersal-extinction cladogenesis (DEC) model using the maximum clade credibility tree from divergence dating analysis based on 25 plastid genes and an uncorrelated molecular clock model under the birth-death tree prior. a, Euronotian region; b, southwest Australia; c, Eremaean region (limited to inland eastern Australia); d, northern region (subregion Atherton); e, Lord Howe Island; f, New Zealand, and g, New Caledonia. Posterior probabilities rounded to the last two decimals and biogeographic ranges that received values below 0.005 not shown. Posterior probabilities >0.50 shaded in dark grey, posterior probabilities >0.20-0.50 shaded in light grey. The chronogram with node numbers is provided in Supplementary Material S.4.3.

Node	a	b	c	f	ab	ac	ad	ae	af	ag	bc	abc	abf
98	0.72	0.00	0.00	0.00	0.17	0.01	0.00	0.00	0.01	0.04	0.00	0.00	0.01
99	0.75	0.00	0.00	0.00	0.14	0.00	0.00	0.00	0.02	0.04	0.00	0.00	0.01
100	0.59	0.01	0.00	0.00	0.39	0.00	0.00	0.00	0.00	0.00	0.00	0.00	0.00
101	0.00	0.10	0.00	0.00	0.89	0.00	0.00	0.00	0.00	0.00	0.00	0.00	0.00
102	0.35	0.01	0.00	0.00	0.63	0.00	0.00	0.00	0.00	0.00	0.00	0.00	0.00
103	0.92	0.00	0.00	0.00	0.07	0.00	0.00	0.00	0.00	0.00	0.00	0.00	0.00
104	0.88	0.00	0.00	0.00	0.10	0.02	0.00	0.00	0.00	0.00	0.00	0.01	0.00
105	0.28	0.00	0.00	0.00	0.35	0.08	0.00	0.00	0.00	0.00	0.00	0.29	0.00
106	0.92	0.00	0.00	0.00	0.00	0.08	0.00	0.00	0.00	0.00	0.00	0.00	0.00
107	0.94	0.00	0.00	0.00	0.00	0.06	0.00	0.00	0.00	0.00	0.00	0.00	0.00
108	0.77	0.00	0.00	0.00	0.00	0.23	0.00	0.00	0.00	0.00	0.00	0.00	0.00
109	0.72	0.00	0.01	0.00	0.00	0.28	0.00	0.00	0.00	0.00	0.00	0.00	0.00
110	0.88	0.00	0.00	0.00	0.00	0.12	0.00	0.00	0.00	0.00	0.00	0.00	0.00
111	0.75	0.00	0.00	0.00	0.00	0.25	0.00	0.00	0.00	0.00	0.00	0.00	0.00
112	0.00	0.00	0.00	0.00	0.00	1.00	0.00	0.00	0.00	0.00	0.00	0.00	0.00
113	0.84	0.00	0.00	0.00	0.00	0.16	0.00	0.00	0.00	0.00	0.00	0.00	0.00
114	1.00	0.00	0.00	0.00	0.00	0.00	0.00	0.00	0.00	0.00	0.00	0.00	0.00
115	1.00	0.00	0.00	0.00	0.00	0.00	0.00	0.00	0.00	0.00	0.00	0.00	0.00
116	0.00	0.00	0.00	0.00	0.51	0.00	0.00	0.00	0.00	0.00	0.05	0.44	0.00
117	0.21	0.00	0.03	0.00	0.00	0.75	0.00	0.00	0.00	0.00	0.00	0.00	0.00
118	0.11	0.00	0.26	0.00	0.00	0.63	0.00	0.00	0.00	0.00	0.00	0.00	0.00
119	0.00	0.00	0.47	0.00	0.00	0.53	0.00	0.00	0.00	0.00	0.00	0.00	0.00
120	0.01	0.00	0.00	0.00	0.00	0.99	0.00	0.00	0.00	0.00	0.00	0.00	0.00
121	1.00	0.00	0.00	0.00	0.00	0.00	0.00	0.00	0.00	0.00	0.00	0.00	0.00
122	0.00	1.00	0.00	0.00	0.00	0.00	0.00	0.00	0.00	0.00	0.00	0.00	0.00
123	0.97	0.00	0.00	0.00	0.03	0.00	0.00	0.00	0.00	0.00	0.00	0.00	0.00
124	0.89	0.00	0.00	0.00	0.11	0.00	0.00	0.00	0.00	0.00	0.00	0.00	0.00
125	0.00	0.19	0.00	0.00	0.81	0.00	0.00	0.00	0.00	0.00	0.00	0.00	0.00
126	0.00	0.16	0.00	0.00	0.84	0.00	0.00	0.00	0.00	0.00	0.00	0.00	0.00
127	0.72	0.00	0.00	0.00	0.28	0.00	0.00	0.00	0.00	0.00	0.00	0.00	0.00
128	1.00	0.00	0.00	0.00	0.00	0.00	0.00	0.00	0.00	0.00	0.00	0.00	0.00
129	1.00	0.00	0.00	0.00	0.00	0.00	0.00	0.00	0.00	0.00	0.00	0.00	0.00
130	1.00	0.00	0.00	0.00	0.00	0.00	0.00	0.00	0.00	0.00	0.00	0.00	0.00
131	1.00	0.00	0.00	0.00	0.00	0.00	0.00	0.00	0.00	0.00	0.00	0.00	0.00
132	1.00	0.00	0.00	0.00	0.00	0.00	0.00	0.00	0.00	0.00	0.00	0.00	0.00
133	1.00	0.00	0.00	0.00	0.00	0.00	0.00	0.00	0.00	0.00	0.00	0.00	0.00
134	1.00	0.00	0.00	0.00	0.00	0.00	0.00	0.00	0.00	0.00	0.00	0.00	0.00
135	1.00	0.00	0.00	0.00	0.00	0.00	0.00	0.00	0.00	0.00	0.00	0.00	0.00
136	0.00	0.13	0.00	0.00	0.87	0.00	0.00	0.00	0.00	0.00	0.00	0.00	0.00
137	0.00	0.00	0.00	0.00	1.00	0.00	0.00	0.00	0.00	0.00	0.00	0.00	0.00
138	0.00	1.00	0.00	0.00	0.00	0.00	0.00	0.00	0.00	0.00	0.00	0.00	0.00
139	0.53	0.00	0.00	0.00	0.00	0.00	0.00	0.00	0.09	0.29	0.00	0.00	0.00
140	0.00	0.00	0.00	0.00	0.00	0.00	0.00	0.00	0.00	0.91	0.00	0.00	0.00
141	0.90	0.00	0.00	0.00	0.00	0.01	0.02	0.00	0.01	0.00	0.00	0.00	0.00
142	0.85	0.00	0.00	0.00	0.00	0.02	0.05	0.00	0.02	0.00	0.00	0.00	0.00
143	0.86	0.00	0.00	0.00	0.00	0.02	0.02	0.00	0.02	0.00	0.00	0.00	0.00
144	0.28	0.00	0.00	0.00	0.00	0.09	0.09	0.00	0.09	0.00	0.00	0.00	0.00
145	0.93	0.00	0.00	0.00	0.00	0.01	0.05	0.00	0.00	0.00	0.00	0.00	0.00
146	0.94	0.00	0.00	0.00	0.00	0.05	0.00	0.01	0.00	0.00	0.00	0.00	0.00
147	0.81	0.00	0.00	0.00	0.00	0.00	0.00	0.19	0.00	0.00	0.00	0.00	0.00
148	0.62	0.00	0.00	0.00	0.00	0.00	0.00	0.00	0.38	0.00	0.00	0.00	0.00
149	0.00	0.00	0.00	0.05	0.00	0.00	0.00	0.00	0.95	0.00	0.00	0.00	0.00

Node	abg	acd	acf	acg	adf	adg	afg	acdf	acdg	acfg	adfg	acdef
98	0.01	0.00	0.00	0.00	0.00	0.00	0.00	0.00	0.00	0.00	0.00	0.00
99	0.01	0.00	0.00	0.00	0.00	0.00	0.00	0.00	0.00	0.00	0.00	0.00
100	0.00	0.00	0.00	0.00	0.00	0.00	0.00	0.00	0.00	0.00	0.00	0.00
101	0.00	0.00	0.00	0.00	0.00	0.00	0.00	0.00	0.00	0.00	0.00	0.00
102	0.00	0.00	0.00	0.00	0.00	0.00	0.00	0.00	0.00	0.00	0.00	0.00
103	0.00	0.00	0.00	0.00	0.00	0.00	0.00	0.00	0.00	0.00	0.00	0.00
104	0.00	0.00	0.00	0.00	0.00	0.00	0.00	0.00	0.00	0.00	0.00	0.00
105	0.00	0.00	0.00	0.00	0.00	0.00	0.00	0.00	0.00	0.00	0.00	0.00
106	0.00	0.00	0.00	0.00	0.00	0.00	0.00	0.00	0.00	0.00	0.00	0.00
107	0.00	0.00	0.00	0.00	0.00	0.00	0.00	0.00	0.00	0.00	0.00	0.00
108	0.00	0.00	0.00	0.00	0.00	0.00	0.00	0.00	0.00	0.00	0.00	0.00
109	0.00	0.00	0.00	0.00	0.00	0.00	0.00	0.00	0.00	0.00	0.00	0.00
110	0.00	0.00	0.00	0.00	0.00	0.00	0.00	0.00	0.00	0.00	0.00	0.00
111	0.00	0.00	0.00	0.00	0.00	0.00	0.00	0.00	0.00	0.00	0.00	0.00
112	0.00	0.00	0.00	0.00	0.00	0.00	0.00	0.00	0.00	0.00	0.00	0.00
113	0.00	0.00	0.00	0.00	0.00	0.00	0.00	0.00	0.00	0.00	0.00	0.00
114	0.00	0.00	0.00	0.00	0.00	0.00	0.00	0.00	0.00	0.00	0.00	0.00
115	0.00	0.00	0.00	0.00	0.00	0.00	0.00	0.00	0.00	0.00	0.00	0.00
116	0.00	0.00	0.00	0.00	0.00	0.00	0.00	0.00	0.00	0.00	0.00	0.00
117	0.00	0.00	0.00	0.00	0.00	0.00	0.00	0.00	0.00	0.00	0.00	0.00
118	0.00	0.00	0.00	0.00	0.00	0.00	0.00	0.00	0.00	0.00	0.00	0.00
119	0.00	0.00	0.00	0.00	0.00	0.00	0.00	0.00	0.00	0.00	0.00	0.00
120	0.00	0.00	0.00	0.00	0.00	0.00	0.00	0.00	0.00	0.00	0.00	0.00
121	0.00	0.00	0.00	0.00	0.00	0.00	0.00	0.00	0.00	0.00	0.00	0.00
122	0.00	0.00	0.00	0.00	0.00	0.00	0.00	0.00	0.00	0.00	0.00	0.00
123	0.00	0.00	0.00	0.00	0.00	0.00	0.00	0.00	0.00	0.00	0.00	0.00
124	0.00	0.00	0.00	0.00	0.00	0.00	0.00	0.00	0.00	0.00	0.00	0.00
125	0.00	0.00	0.00	0.00	0.00	0.00	0.00	0.00	0.00	0.00	0.00	0.00
126	0.00	0.00	0.00	0.00	0.00	0.00	0.00	0.00	0.00	0.00	0.00	0.00
127	0.00	0.00	0.00	0.00	0.00	0.00	0.00	0.00	0.00	0.00	0.00	0.00
128	0.00	0.00	0.00	0.00	0.00	0.00	0.00	0.00	0.00	0.00	0.00	0.00
129	0.00	0.00	0.00	0.00	0.00	0.00	0.00	0.00	0.00	0.00	0.00	0.00
130	0.00	0.00	0.00	0.00	0.00	0.00	0.00	0.00	0.00	0.00	0.00	0.00
131	0.00	0.00	0.00	0.00	0.00	0.00	0.00	0.00	0.00	0.00	0.00	0.00
132	0.00	0.00	0.00	0.00	0.00	0.00	0.00	0.00	0.00	0.00	0.00	0.00
133	0.00	0.00	0.00	0.00	0.00	0.00	0.00	0.00	0.00	0.00	0.00	0.00
134	0.00	0.00	0.00	0.00	0.00	0.00	0.00	0.00	0.00	0.00	0.00	0.00
135	0.00	0.00	0.00	0.00	0.00	0.00	0.00	0.00	0.00	0.00	0.00	0.00
136	0.00	0.00	0.00	0.00	0.00	0.00	0.00	0.00	0.00	0.00	0.00	0.00
137	0.00	0.00	0.00	0.00	0.00	0.00	0.00	0.00	0.00	0.00	0.00	0.00
138	0.00	0.00	0.00	0.00	0.00	0.00	0.00	0.00	0.00	0.00	0.00	0.00
139	0.00	0.00	0.00	0.01	0.00	0.01	0.03	0.00	0.01	0.01	0.01	0.00
140	0.00	0.00	0.00	0.01	0.00	0.02	0.01	0.00	0.01	0.01	0.01	0.00
141	0.00	0.01	0.01	0.00	0.01	0.00	0.00	0.01	0.00	0.00	0.00	0.00
142	0.00	0.01	0.01	0.00	0.01	0.00	0.00	0.03	0.00	0.00	0.00	0.00
143	0.00	0.01	0.01	0.00	0.01	0.00	0.00	0.03	0.00	0.00	0.00	0.00
144	0.00	0.09	0.09	0.00	0.09	0.00	0.00	0.09	0.00	0.00	0.00	0.09
145	0.00	0.00	0.00	0.00	0.00	0.00	0.00	0.00	0.00	0.00	0.00	0.00
146	0.00	0.00	0.00	0.00	0.00	0.00	0.00	0.00	0.00	0.00	0.00	0.00
147	0.00	0.00	0.00	0.00	0.00	0.00	0.00	0.00	0.00	0.00	0.00	0.00
148	0.00	0.00	0.00	0.00	0.00	0.00	0.00	0.00	0.00	0.00	0.00	0.00
149	0.00	0.00	0.00	0.00	0.00	0.00	0.00	0.00	0.00	0.00	0.00	0.00

Supplementary Material S4.2. Continued.

Node #	a	b	c	f	ab	ac	ad	ae	af	ag	bc	abc	abf	abg	acd	acf	acg	adf	adg	afg	acdf	acdg	acfg	adfg	acdef
150	0.17	0.00	0.00	0.00	0.00	0.00	0.00	0.00	0.83	0.00	0.00	0.00	0.00	0.00	0.00	0.00	0.00	0.00	0.00	0.00	0.00	0.00	0.00	0.00	0.00
151	1.00	0.00	0.00	0.00	0.00	0.00	0.00	0.00	0.00	0.00	0.00	0.00	0.00	0.00	0.00	0.00	0.00	0.00	0.00	0.00	0.00	0.00	0.00	0.00	0.00
152	1.00	0.00	0.00	0.00	0.00	0.00	0.00	0.00	0.00	0.00	0.00	0.00	0.00	0.00	0.00	0.00	0.00	0.00	0.00	0.00	0.00	0.00	0.00	0.00	0.00
153	1.00	0.00	0.00	0.00	0.00	0.00	0.00	0.00	0.00	0.00	0.00	0.00	0.00	0.00	0.00	0.00	0.00	0.00	0.00	0.00	0.00	0.00	0.00	0.00	0.00
154	0.00	0.00	0.00	1.00	0.00	0.00	0.00	0.00	0.00	0.00	0.00	0.00	0.00	0.00	0.00	0.00	0.00	0.00	0.00	0.00	0.00	0.00	0.00	0.00	0.00
155	0.00	0.00	0.00	1.00	0.00	0.00	0.00	0.00	0.00	0.00	0.00	0.00	0.00	0.00	0.00	0.00	0.00	0.00	0.00	0.00	0.00	0.00	0.00	0.00	0.00
156	0.00	0.00	0.00	1.00	0.00	0.00	0.00	0.00	0.00	0.00	0.00	0.00	0.00	0.00	0.00	0.00	0.00	0.00	0.00	0.00	0.00	0.00	0.00	0.00	0.00
157	1.00	0.00	0.00	0.00	0.00	0.00	0.00	0.00	0.00	0.00	0.00	0.00	0.00	0.00	0.00	0.00	0.00	0.00	0.00	0.00	0.00	0.00	0.00	0.00	0.00
158	0.84	0.00	0.00	0.00	0.14	0.01	0.00	0.00	0.00	0.00	0.00	0.01	0.00	0.00	0.00	0.00	0.00	0.00	0.00	0.00	0.00	0.00	0.00	0.00	0.00
159	0.55	0.00	0.00	0.00	0.41	0.01	0.00	0.00	0.00	0.00	0.00	0.02	0.00	0.00	0.00	0.00	0.00	0.00	0.00	0.00	0.00	0.00	0.00	0.00	0.00
160	0.81	0.00	0.00	0.00	0.18	0.00	0.00	0.00	0.00	0.01	0.00	0.00	0.00	0.00	0.00	0.00	0.00	0.00	0.00	0.00	0.00	0.00	0.00	0.00	0.00
161	0.97	0.00	0.00	0.00	0.00	0.00	0.00	0.00	0.01	0.03	0.00	0.00	0.00	0.00	0.00	0.00	0.00	0.00	0.00	0.00	0.00	0.00	0.00	0.00	0.00
162	0.92	0.00	0.00	0.00	0.00	0.00	0.00	0.00	0.08	0.00	0.00	0.00	0.00	0.00	0.00	0.00	0.00	0.00	0.00	0.00	0.00	0.00	0.00	0.00	0.00
163	1.00	0.00	0.00	0.00	0.00	0.00	0.00	0.00	0.00	0.00	0.00	0.00	0.00	0.00	0.00	0.00	0.00	0.00	0.00	0.00	0.00	0.00	0.00	0.00	0.00
164	1.00	0.00	0.00	0.00	0.00	0.00	0.00	0.00	0.00	0.00	0.00	0.00	0.00	0.00	0.00	0.00	0.00	0.00	0.00	0.00	0.00	0.00	0.00	0.00	0.00
165	1.00	0.00	0.00	0.00	0.00	0.00	0.00	0.00	0.00	0.00	0.00	0.00	0.00	0.00	0.00	0.00	0.00	0.00	0.00	0.00	0.00	0.00	0.00	0.00	0.00
166	1.00	0.00	0.00	0.00	0.00	0.00	0.00	0.00	0.00	0.00	0.00	0.00	0.00	0.00	0.00	0.00	0.00	0.00	0.00	0.00	0.00	0.00	0.00	0.00	0.00
167	1.00	0.00	0.00	0.00	0.00	0.00	0.00	0.00	0.00	0.00	0.00	0.00	0.00	0.00	0.00	0.00	0.00	0.00	0.00	0.00	0.00	0.00	0.00	0.00	0.00
168	1.00	0.00	0.00	0.00	0.00	0.00	0.00	0.00	0.00	0.00	0.00	0.00	0.00	0.00	0.00	0.00	0.00	0.00	0.00	0.00	0.00	0.00	0.00	0.00	0.00
169	1.00	0.00	0.00	0.00	0.00	0.00	0.00	0.00	0.00	0.00	0.00	0.00	0.00	0.00	0.00	0.00	0.00	0.00	0.00	0.00	0.00	0.00	0.00	0.00	0.00
170	1.00	0.00	0.00	0.00	0.00	0.00	0.00	0.00	0.00	0.00	0.00	0.00	0.00	0.00	0.00	0.00	0.00	0.00	0.00	0.00	0.00	0.00	0.00	0.00	0.00
171	1.00	0.00	0.00	0.00	0.00	0.00	0.00	0.00	0.00	0.00	0.00	0.00	0.00	0.00	0.00	0.00	0.00	0.00	0.00	0.00	0.00	0.00	0.00	0.00	0.00
172	1.00	0.00	0.00	0.00	0.00	0.00	0.00	0.00	0.00	0.00	0.00	0.00	0.00	0.00	0.00	0.00	0.00	0.00	0.00	0.00	0.00	0.00	0.00	0.00	0.00
173	1.00	0.00	0.00	0.00	0.00	0.00	0.00	0.00	0.00	0.00	0.00	0.00	0.00	0.00	0.00	0.00	0.00	0.00	0.00	0.00	0.00	0.00	0.00	0.00	0.00
174	0.00	0.00	0.00	0.00	0.00	0.00	0.00	0.00	1.00	0.00	0.00	0.00	0.00	0.00	0.00	0.00	0.00	0.00	0.00	0.00	0.00	0.00	0.00	0.00	0.00
175	0.00	0.00	0.00	0.00	0.00	0.00	0.00	0.00	0.00	1.00	0.00	0.00	0.00	0.00	0.00	0.00	0.00	0.00	0.00	0.00	0.00	0.00	0.00	0.00	0.00
176	1.00	0.00	0.00	0.00	0.00	0.00	0.00	0.00	0.00	0.00	0.00	0.00	0.00	0.00	0.00	0.00	0.00	0.00	0.00	0.00	0.00	0.00	0.00	0.00	0.00
177	0.37	0.00	0.00	0.00	0.62	0.00	0.00	0.00	0.00	0.00	0.00	0.00	0.00	0.00	0.00	0.00	0.00	0.00	0.00	0.00	0.00	0.00	0.00	0.00	0.00
178	0.00	0.37	0.00	0.00	0.63	0.00	0.00	0.00	0.00	0.00	0.00	0.00	0.00	0.00	0.00	0.00	0.00	0.00	0.00	0.00	0.00	0.00	0.00	0.00	0.00
179	0.00	0.34	0.00	0.00	0.64	0.00	0.00	0.00	0.00	0.00	0.00	0.02	0.00	0.00	0.00	0.00	0.00	0.00	0.00	0.00	0.00	0.00	0.00	0.00	0.00
180	0.02	0.00	0.00	0.00	0.88	0.03	0.00	0.00	0.00	0.00	0.00	0.07	0.00	0.00	0.00	0.00	0.00	0.00	0.00	0.00	0.00	0.00	0.00	0.00	0.00
181	0.00	1.00	0.00	0.00	0.00	0.00	0.00	0.00	0.00	0.00	0.00	0.00	0.00	0.00	0.00	0.00	0.00	0.00	0.00	0.00	0.00	0.00	0.00	0.00	0.00
182	0.00	0.00	0.00	0.00	0.94	0.00	0.00	0.00	0.00	0.00	0.00	0.06	0.00	0.00	0.00	0.00	0.00	0.00	0.00	0.00	0.00	0.00	0.00	0.00	0.00
183	0.93	0.00	0.00	0.00	0.00	0.07	0.00	0.00	0.00	0.00	0.00	0.00	0.00	0.00	0.00	0.00	0.00	0.00	0.00	0.00	0.00	0.00	0.00	0.00	0.00
184	0.69	0.00	0.00	0.00	0.00	0.31	0.00	0.00	0.00	0.00	0.00	0.00	0.00	0.00	0.00	0.00	0.00	0.00	0.00	0.00	0.00	0.00	0.00	0.00	0.00
185	1.00	0.00	0.00	0.00	0.00	0.00	0.00	0.00	0.00	0.00	0.00	0.00	0.00	0.00	0.00	0.00	0.00	0.00	0.00	0.00	0.00	0.00	0.00	0.00	0.00
186	0.49	0.00	0.00	0.00	0.00	0.51	0.00	0.00	0.00	0.00	0.00	0.00	0.00	0.00	0.00	0.00	0.00	0.00	0.00	0.00	0.00	0.00	0.00	0.00	0.00
187	1.00	0.00	0.00	0.00	0.00	0.00	0.00	0.00	0.00	0.00	0.00	0.00	0.00	0.00	0.00	0.00	0.00	0.00	0.00	0.00	0.00	0.00	0.00	0.00	0.00
188	1.00	0.00	0.00	0.00	0.00	0.00	0.00	0.00	0.00	0.00	0.00	0.00	0.00	0.00	0.00	0.00	0.00	0.00	0.00	0.00	0.00	0.00	0.00	0.00	0.00
189	1.00	0.00	0.00	0.00	0.00	0.00	0.00	0.00	0.00	0.00	0.00	0.00	0.00	0.00	0.00	0.00	0.00	0.00	0.00	0.00	0.00	0.00	0.00	0.00	0.00
190	1.00	0.00	0.00	0.00	0.00	0.00	0.00	0.00	0.00	0.00	0.00	0.00	0.00	0.00	0.00	0.00	0.00	0.00	0.00	0.00	0.00	0.00	0.00	0.00	0.00
191	1.00	0.00	0.00	0.00	0.00	0.00	0.00	0.00	0.00	0.00	0.00	0.00	0.00	0.00	0.00	0.00	0.00	0.00	0.00	0.00	0.00	0.00	0.00	0.00	0.00
192	1.00	0.00	0.00	0.00	0.00	0.00	0.00	0.00	0.00	0.00	0.00	0.00	0.00	0.00	0.00	0.00	0.00	0.00	0.00	0.00	0.00	0.00	0.00	0.00	0.00
193	1.00	0.00	0.00	0.00	0.00	0.00	0.00	0.00	0.00	0.00	0.00	0.00	0.00	0.00	0.00	0.00	0.00	0.00	0.00	0.00	0.00	0.00	0.00	0.00	0.00

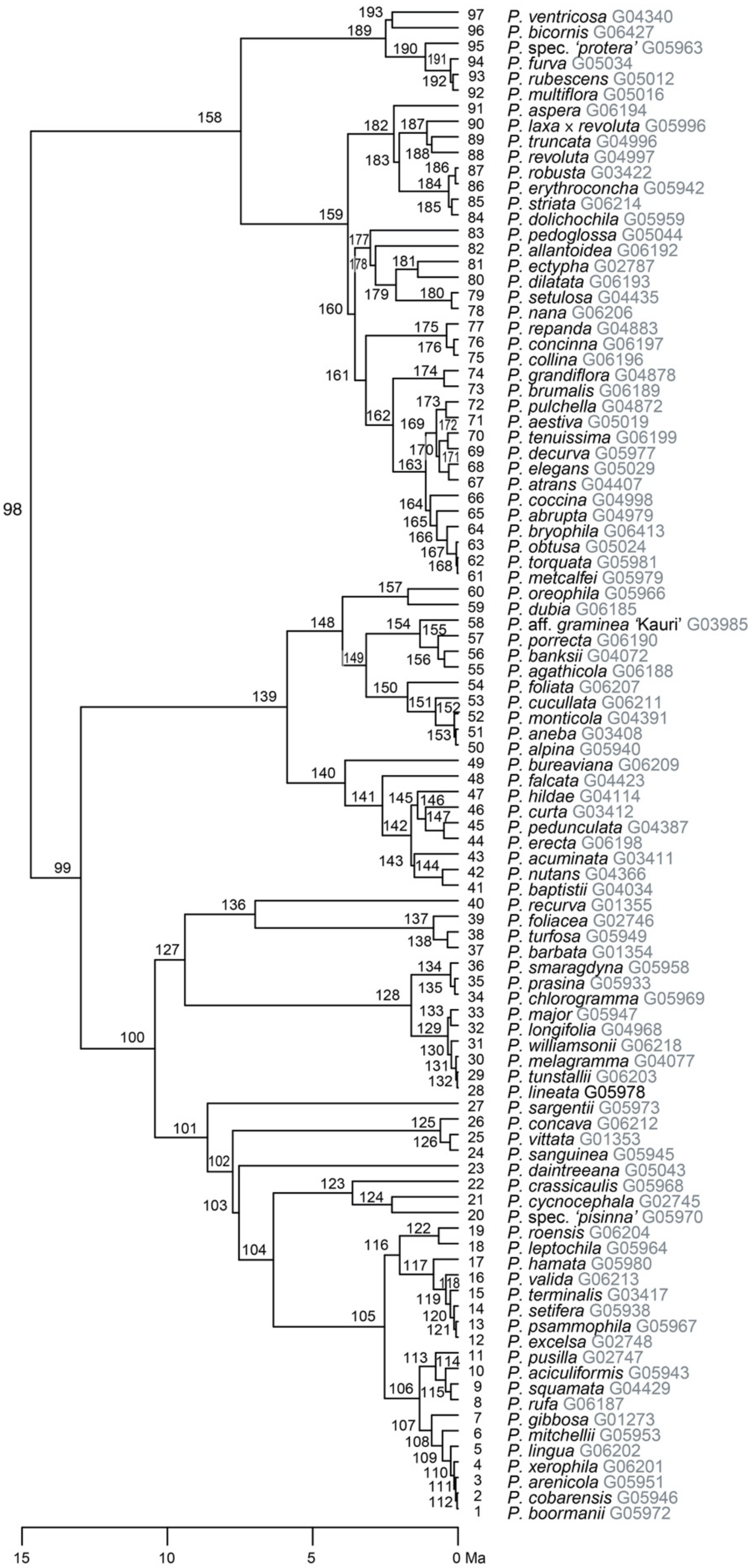

Supplementary Material S4.3. Node numbers of the maximum clade credibility tree from divergence time estimation in Pterostylidinae based on 25 plastid genes under the birth-death tree prior and uncorrelated clock model used for ancestral range analysis. Nodes and tips are numbered consecutively. Ma: Million years; G-number: sample identifier.
